# To Regulate the Practice

**Published:** 1896-02

**Authors:** 


TO REGULATE THE PRACTICE.—At a meeting of the East Texas Medical Association, held at Tyler, Texas, Jan. 15, 1896, Dr. E. A. Woldert read a paper, entitled "A Plea for a More Efficient Law Governing the Practice of Medicine."* He presented at the same time the draft of a bill for the purpose. It was discussed and amended by the members, and, with a suitable preamble and resolutions, was adopted by unanimous vote, as expressing the sentiments of the Asssociation.

It is the wish and intention of the promoters of this scheme to submit this bill to the committee appointed by the Texas State Medical Association to do similar work, and which committee consists of Drs. T. J. Wilson, Sherman; J. F. Y. Paine, Galveston; B. Saunders, of Fort Worth; M. D. Knox, Hillsboro, and T. D. Wooten, Austin, for their approval or for amendment, and then lay it before the State Medical Association at its next meeting, which will be held at Fort Worth the last week in April next. As amended and perfected then and there, we learn, it is to be presented to the next legislature and its passage urged.

The bill, as at present drawn, defines the meaning of "Practice of Medicine." It provides for three separate boards of examiners, one board for each so-called "school," *i. e.*, one for physicians, one for homeopaths, and one for eclectics; applicants of each pursuasion to be examined by their appropriate board. The Regents of the State University are to be at the head of the whole, and are to appoint the members of the boards, five to each board—selected from lists of ten or more names submitted by the respective State associations. The Regents are to give each applicant a preliminary examination, and are to be the judge of the diploma presented, if the applicant be a graduate. It must be a *bona fide* diploma, and emanating from a school of known respectability. The most fatal defect of the existing law is—that any kind of a diploma—from anywhere—can be recorded

---

[*This paper will be published in our next issue.—ED.]

and must be accepted, virtually, as license to practice; it is made the business of no one to discriminate in this regard. When the Regents have satisfied themselves that the applicant possesses the necessary qualification to entitle him to an examination, they give him an order to the board, or issue a permit for examination. Each State Medical Association is to prepare a set or sets of questions, and furnish them to the Regents, who are to select such questions from each list as are to be propounded to the applicant. A fee of $15 is to be paid for each examination.

The above are the principal features of the bill, as it now stands. What it will be when it has passed through the hands of the State Medical Association, and its committee, is further along. The East Texas Medical Association wish it to be distinctly understood that in this matter they earnestly desire the aid and co-operation of the said committee, and that they are not trying to take the matter out of their hands by this move. It is hoped that this draft will serve the committee at least as a basis, and save them some trouble. The bill was drawn up by Dr. Woldert and his colleagues, after carefully examining the laws of several States, and it embodies the good features, they say, of especially the law in New York, which has been found to work very successfnlly.

Publication in the present shape would be, we believe, premature and useless, and criticism at our hands would also be out of place just yet. When the draft has been perfected; when it shall have been gotten into the shape in which it will be presented to the legislature, the JOURNAL will publish it with pleasure, and, at the same time, give our views upon its merits and defects—if any defects; and to its passage we pledge our support and best efforts.

